# Crosstalk between autophagy and immune cell infiltration in the tumor microenvironment

**DOI:** 10.3389/fmed.2023.1125692

**Published:** 2023-02-06

**Authors:** Tiantian Yang, Yang Zhang, Junhang Chen, Longhao Sun

**Affiliations:** Department of General Surgery, Tianjin Medical University General Hospital, Tianjin, China

**Keywords:** autophagy, tumor immunity, dendritic cell, macrophage, immune cell infiltration

## Abstract

Autophagy is a conserved process for self-degradation and provides cells with a rescue mechanism to respond to circumstances such as stress and starvation. The role of autophagy in cancer is extremely complex and often paradoxical. Most of the related published studies on tumors are always focused on cancer cells. However, present studies gradually noticed the significance of autophagy in the tumor microenvironment. These studies demonstrate that autophagy and immunity work synergistically to affect tumor progression, indicating that autophagy could become a potential target for cancer immunotherapy. Therefore, it is crucial to clarify the correlation between autophagy and various tumor-infiltrating immune cells in the tumor microenvironment. The context-dependent role of autophagy is critical in the design of therapeutic strategies for cancer.

## Introduction

Autophagy is a conserved process for self-degradation by which misfolded cytosolic proteins and damaged organelles are sequestered in intracellular double-membrane vesicles and delivered to lysosomes (1, 2). This process usually happens under circumstances such as stress and starvation to provide cells with a rescue mechanism. Currently, autophagy is usually classified into three subtypes such as macroautophagy, minorautophagy, and chaperone-mediated autophagy (3), and the first subtype is the most studied one and will be the subject of discussion in this study. Numerous studies unveiled that macro-autophagy/autophagy is a crucial homeostatic process for the regulation of biological activities in both physiological and pathophysiological statuses (4, 5). In addition, published articles also found that autophagy dysfunction is associated with various diseases, and increasing evidence highlighted the essential role of autophagy in cancer (6, 7). However, the current understanding of the specific mechanism of autophagy in tumor immunity is quite limited. This study will describe a comprehensive complex relationship between autophagy and cancer, especially the impact on the tumor immune microenvironment, and explore the promising prospect of autophagy manipulation as a potential approach to improve anticancer therapeutics.

## Autophagy and tumor

**Figure 1:**
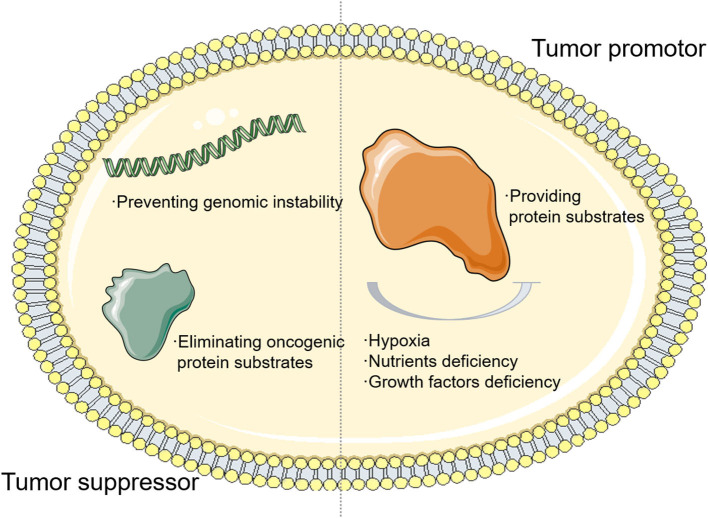
The relationship between autophagy and tumor. The autophagy signaling pathway, which is usually deprived of nutrients, oxygen, and growth factor, contributes to tumor cell homeostasis and rapid adaptation to environmental changes.

As an adaptive process responding to cellular microenvironment changes, the function of autophagy in a tumor is complicated and volatile depending on the cellular context. First, autophagy could prevent genomic instability and eliminate oncogenic protein substrates, acting as a tumor suppressor. The absence of beclin 1 (BECN1), one of the autophagy genes, is observed to widely occur in human cancer cases (8–10). Meanwhile, as a Becn1-binding autophagy regulator, the ultraviolet radiation resistance-associated gene (UVRAG) shows non-sense mutations in some gastric cancer cases, suggesting that autophagy may suppress tumor initiation (11, 12). On the contrary, autophagy can function as a tumor promoter by providing substrates under hypoxia or nutrient deficiency. The autophagy signaling pathway, which is usually deprived of nutrients, oxygen, and growth factor (13, 14), contributes to tumor cell homeostasis and rapid adaptation to environmental changes (Figure 1). For example, previous studies pointed out that tumor cells that lack autophagy-related genes are more sensitive to metabolic alteration (15, 16). All these factors indicate that autophagy facilitates tumorigenesis in advanced tumors and autophagy could become a novel cancer therapeutic target for clinical treatment.

## Autophagy and tumor immune cell infiltration

Autophagy functions in an extremely intricate manner in cancer due to diverse tumor microenvironments (TMEs). Most of the previous autophagy-related studies on tumors focused only on cancer cells (17, 18). Solid tumors also contain various stromal components, including fibroblasts, endothelial cells, and particularly tumor-infiltrating immune cells. As two inseparable entities, varieties of immune mediators modulate the autophagy in TME, and the immune signaling cascades are also mediated by autophagy at the same time (19, 20). This harmonious co-adjustment mechanism maintains the homeostasis of a human innate immune response. The roles of autophagy in regulating tumor immunity and tumor-infiltrating immune cells range from tumor antigen recognition and presentation by antigen-presenting cells (APCs) to T-cell receptor (TCR)-specific lymphocyte activation and development (21, 22).

In recent decades, current studies noticed the significance of autophagy in the TME gradually, demonstrating that autophagy and immunity work synergistically to affect tumor progression, indicating that autophagy could become a potential target for cancer immunotherapy (23, 24). Therefore, it is necessary to clarify the correlation between autophagy and various tumor-infiltrating immune cells in the TME and broaden new insights into cancer therapy.

### Macrophage

**Figure 2:**
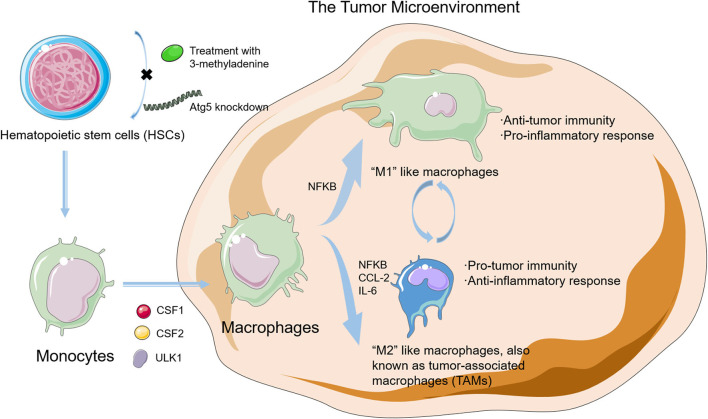
The differentiation in macrophages and the relationship between autophagy and macrophages in the tumor microenvironment (TME). Autophagy plays a vital role in the differentiation and polarization of macrophage in tumor tissues, which indicates intimate links among cancer, autophagy, and macrophage.

Macrophages, as a type of differentiated leukocyte which is responsible for homeostasis, are of the highest concentration in a TME (25, 26). Macrophages are derived from hematopoietic stem cells (HSCs) in the bone marrow and differentiate into monocytes that are short-lived and programmed to undergo apoptosis without stimulation in blood. Under such circumstances, however, monocytes would activate survival pathways and recruit into the tumor tissue and differentiate into macrophages. Macrophages could be classified into two subtypes. “M1-like” phenotype macrophages are associated with anticancer immunity and a pro-inflammatory effect. “M2-like” phenotype macrophages are associated with immunosuppression and an anti-inflammatory effect. These two subtypes could exert antagonistic functions according to different TMEs (27). Furthermore, according to the alteration in TME, two fully polarized subgroups can repolarize and transform mutually (28, 29). Tumor-associated macrophages (TAMs), also known as tumor-infiltrating macrophages, mainly represent the M2-like phenotype and facilitate tumor progression through their potent immunosuppressive activities. Because of their short half-lives, TAMs need to be supplied ceaselessly. Increasing bodies of evidence demonstrate that autophagy plays a vital role in the differentiation and polarization of macrophages in tumor tissues, which indicates the intimate links among cancer, autophagy, and macrophage (Figure 2) (30, 31).

Current studies point out that autophagy plays a leading part in HSC self-renewal, maintenance, and differentiation (32, 33). Evidence also proves that 3-methyladenine (3-MA) treatment or autophagy-related protein 5 (ATG5) knockdown could inhibit autophagy by blocking the self-renewal and differentiation in HSCs (34). Under metabolic stress conditions, HSCs could exhibit an intact forkhead box class O3a (FOXO3a)-induced pro-autophagic gene program to induce autophagy to protect HSCs against apoptosis, thus highlighting autophagy as a critical pathway in the HSC maintenance process (35).

Tumor-associated macrophages originate from monocytes and then recruit into the tumor site through several chemokines and cytokines. As one of the most prominent chemoattractants in the recruitment of monocytes, chemokine (C–C motif) ligand 2 (CCL2) also helps the monocytes overexpress antiapoptotic proteins, inhibit caspase-8 cleavage, and upregulate autophagy to protect them against apoptosis (36, 37). Recombinant capsid viral protein 1 (rVP1) could induce apoptosis, modulate the CCL2 production, and then inhibit the proliferation and metastasis of tumor cells (38). Furthermore, rVP1 also upregulates autophagy in tumor cells by increasing the phosphorylation levels of mitogen-activated protein kinase 3 (MAPK3)/extracellular signal-regulated kinase 1 (ERK1) and mitogen-activated protein kinase 1/extracellular signal-regulated kinase 2 (MAPK1/ERK2) and the activity of matrix metalloproteinase 9 (MMP9) and then promotes the migration of macrophages.

Autophagy also plays a key role in the differentiation from monocytes into macrophages. Colony-stimulating factor 1 (CSF1) could induce the differentiation of monocytes into macrophages, and under this process, the level of unc-51-like autophagy activating kinase 1 (ULK1) expression and phosphorylation increases, which finally activates autophagy (39–41). When autophagy is blockaded by the pharmacological treatment or autophagy-related protein 7 (Atg7) silence, the CSF1-driven macrophage differentiation is hampered significantly. The colony-stimulating factor 2 (CSF2) is another critical cytokine that takes part in the differentiation of monocytes into macrophages, and autophagy is activated during this process. When autophagy is blockaded, the CSF2-induced differentiation process is also inhibited (42).

Autophagy is also involved in the coordination of macrophage polarization. Macrophage polarization in the TME is modulated by polarization-related factors or intracellular signaling mechanisms, such as the nuclear factor of kappa light polypeptide gene enhancer in B cells (NFKB) and the mechanistic target of rapamycin kinase (MTOR). As a transcriptional factor that acts as an essential bridge between inflammation and cancer, NFKB is also involved in the regulation of both M1 and M2 phenotype–macrophage polarization (43). NFKB is an indispensable link in the polarization of M2 macrophages and exhibits a low activity in TAMs. Hepatoma-derived toll-like receptor 2 (TLR2)-related ligands can trigger the M2 macrophage polarization through the RelA/NFKB pathway by autophagy (44, 45). Meanwhile, when inhibiting autophagy pharmacologically or genetically, NFKB activity saves and M2 macrophages repolarize to produce more M1-like cytokines, thereby unleashing the anticancer effects. As a conserved protein kinase regulating autophagy, MTOR is also critical in the modulation of macrophage polarization (46, 47). Rapamycin inhibits the MTOR pathway and causes polarization toward the M1 phenotype; however, knockdown of the MTOR repressor tuberous sclerosis 2 (TSC2) could activate this pathway that exerts an opposite effect (48). In the TME, both CCL2 and interleukin 6 (IL-6) could induce autophagy, inhibit apoptosis in macrophages, and stimulate macrophage polarization toward the M2 phenotype. Moreover, in myeloid cell-specific autophagic-deficient mice, the recruitment of M2-like macrophages to metastatic niches is inhibited by impairing the transforming growth factor-β1 (TGF-β1) signaling pathway that finally suppresses the cancer metastasis. Altogether, these findings demonstrate that the role of autophagy in macrophage polarization and recruitment in the TME is significant.

### Dendritic cell

Similar to most of the pivotal professional APCs, dendritic cells (DCs) are also critical for adaptive anticancer immunity. The DCs recognize and present not only extracellular (exogenous) peptide epitopes by the major histocompatibility complex (MHC) class II to the cluster of differentiation 4 (CD4^+^) T-lymphocytes, but also intracellular (endogenous) peptide epitopes by the MHC class I molecules to the cluster of differentiation 8 (CD8^+^) T-lymphocytes. Reactive oxygen species (ROS) is one of the most potent molecules in impairing the tumor suppressive function of DCs with an abundant presence in the TME (49, 50). High concentrations of ROS activate oxidative stress, resulting in the cell death of DCs mediated by the c-Jun N-terminal kinase (JNK) pathway (51). An autophagy defect would cause an accumulation of ROS and finally hamper the DCs.

In DCs, intracellular antigen routing and presentation could be influenced by autophagy, and the MHC class II to CD4^+^ lymphocyte pathway is preferred. The pharmacological treatment or the silencing of autophagy genes could inhibit autophagy and upregulate the expression of MHC-I on DCs by a slower internalization of MHC-I. Therefore, when there is a lack of autophagy, the expression of MHC-I molecules would be elevated and the degradation is reduced. On the contrary, 3-MA inhibits autophagy and thereby, decreases the expression of MHC-II on DCs. The antigen presentation in DCs is restricted by the immunosuppressive molecule, T-cell immunoglobulin and mucin domain-containing 4 (TIMD4), through an AMP-activated protein kinase (AMPK)-driven autophagic degradation mechanism. Previous studies proved that the expression of regulatory T (Treg) cells is associated with abnormal anticancer immunity and a worse clinical prognosis (52, 53). In reality, Treg inhibits autophagy and suppresses the effective autoimmune response (54). Cytotoxic T lymphocyte-associated protein 4 (CTLA4)-specific immune-checkpoint blockers have been approved for melanoma treatment and derepress autophagic responses of DCs in the Treg-infiltrated TME.

The cross-presentation of DCs is associated with increased levels of autophagy. The MHC-I molecule-mediated tumor antigen cross-presentation process is usually activated by autophagy to generate an effective antitumor cytotoxic T lymphocyte (CTL) response. Autophagy inhibition reduces the MHC-dependent cross-presentation abilities of DCs and consequently downregulates the antigen-specific T-cell responses. Taken together, autophagy inhibition significantly impairs the MHC-dependent tumor antigen presentation and influences antigen-specific T-cell immunity negatively.

### T cell

Autophagy is indispensable for the proliferation and survival of T lymphocytes. Autophagy contributes to the development and effector function of T cells and, thereby, shapes human adaptive immunity (55, 56). Abundant autophagosomes are present in TCR-activated CTLs and T-helper (Th) cell subsets, whereas only a minimal number of autophagosomes are present in naïve T-cells (57–59). T-cell activation by interleukin 2 (IL-2) or 4-1BB signaling causes the induction of autophagy and an increase in lysosomal content. Moreover, T-cell activation also facilitates the fusion process between the autophagosomes and the lysosomes and increases the autophagic flux as a consequence. On the contrary, when knocking out the core genes of autophagy or several autophagy-related upstream regulators, the proliferation of T cells would be impaired due to the activation of TCR, and adding co-stimulation of CD28 or interleukin 2 (IL-2) cannot prevent this impairment process (60, 61). In the autophagy-knockout mouse models, the number of T cells is severely reduced, especially the CTL counts. Knockout of autophagy genes or regulators, such as autophagy-related protein 3 (ATG3), ATG5, or ATG7, results in more active caspase-9 in activated T cells, which indicates a higher level of apoptosis (62). Additionally, once the TCR is activated, T cells lacking in autophagy would secrete fewer pro-inflammatory cytokines, and the survival of T cells could also be influenced negatively (63).

Furthermore, autophagy also mediates the proliferation and memory maintenance of T lymphocytes. In proliferating T cells, autophagy is usually upregulated and determines the survival of T cells (64, 65). When lacking in autophagy, the aging CD8^+^ T cells would cause mitochondrial dysfunction, an elevation of reactive oxygen species (ROS), and an increase in p38 during the terminal differentiation phase. In reality, p38 could suppress autophagy that repairs the proliferative function in these senescent CD8^+^ T cells (66, 67). In all, autophagy plays a major role in the normal functioning of T cells and the formation of memory T cells. Therefore, treating patients with cancer with autophagy inhibitors could impair the function and activity of T cells as well as hamper their anticancer immune response.

Autophagy maintains T-cell homeostasis by degrading mitochondria and proapoptotic proteins. When knocking out ATG5 in T cells, the mitochondrial mass increases and the ROS gets elevated, which suggests that the inhibition of autophagy deregulates the clearance of organelles and breaks the mitochondrial homeostasis (68). However, activating autophagy with an autophagy inducer could reduce the level of ROS and restore T-cell proliferation and survival.

Autophagy directly mediates the expression of apoptotic proteins in T cells, such as the apoptosis-inducing factor (AIF), caspase-3, caspase-8, B-cell lymphoma 2 (Bcl-2), B-cell lymphoma 2-like 11 (BCL2L11, also known as BIM), B-cell lymphoma-extra large (Bcl-xl), and Bcl-2-associated X protein (BAX). For example, the inhibition of autophagy using Beclin-1-knockout increases the expression levels of caspase-3 and−8 proteins (69–71). Conversely, the activation of autophagy by rapamycin downregulates the caspase-3 levels. Meanwhile, autophagy could also influence cell cycle inhibitors such as the cyclin-dependent kinase inhibitor 1B (CDKN1B), which is usually degraded by autophagy (72, 73). When inhibiting autophagy, the degradation of CDKN1B as well as the proliferation of T cells is usually prevented.

Autophagy could also influence T-cell-mediated anticancer immunity. The immune checkpoints (ICPs) are pivotal inhibitors of anticancer immunity in the TME. For example, in lung cancer cells, the inhibitor could activate autophagy and shrink the expression level of programmed death-ligand 1 (PD-L1), while the activation of mTOR may render the opposite effect (74). The treatment with an mTOR inhibitor, such as rapamycin, reduces the expression level of PD-L1 and reactivates anticancer T-cell immunity by inducing autophagy (75, 76). Furthermore, the effect of reducing tumor growth with a combination of rapamycin and PD-L1 is better than the treatment alone.

### Natural killer cell

Autophagy downregulates the sensitivity of cancer cells toward natural killer cell (NK)-mediated cell lysis and results in an impairment of anticancer immunity. High levels of autophagy could affect the stability of the immunological synapse between NK and its target cancer cell, thereby reducing the efficacy of NK-mediated lysis. During the process of NK-mediated lysis, some connexin proteins play a vital role in the exchange of small molecules between the effector and target cells, since these connexin proteins are essential for the formation of gap junctions, especially connexin-43 (77). Under hypoxic conditions, the accumulation of connexin-43 at the immunological synapse of melanoma cells is reduced. Autophagy inhibition as well as the NK-mediated cell lysis will restore the accumulation. Furthermore, as the main cytotoxic molecule, active granzyme B is also transported into the cancer cells by connexin-43 in NK cells (78). Hence, autophagy degrades connexin-43 and influences cytolysis in many different ways. In lung cancer cells, breast cancer cells, and melanoma cells, autophagy is upregulated and lysis resistance is mediated by degrading granzyme B in hypoxic conditions (79–81). To sum up, upregulated autophagy could inhibit the immunological synapse and negatively affect the lysis sensitivity in cancer cells.

### Regulatory T cell

Regulatory T (Treg) cell inhibits anticancer immunity as a subtype of CD4^+^ T cell. Increased Treg infiltration into the tumor bed indicates poor survival (82). Tregs contain increased autophagy than naïve CD4^+^ cells (83). Autophagy silence in Tregs induces apoptosis and blocks Treg-mediated immunosuppression, which subsequently yields increasing CTLs and smaller tumors in colon adenocarcinoma. Therefore, autophagy is critical for Treg-mediated immunosuppression, which could be blocked by autophagy inhibition.

### Autophagy and immunogenic cell death

Autophagy modulates the induction of immunogenic cell death (ICD), a non-silent form of cell death, which activates the development of tumor-eradicating CTL response. In cancer cells, ICD helps to release tumor antigens, which are processed by the APC and ultimately result in the activation of CTL and systemic tumor rejection (84). Numerous conventional cancer therapeutic strategies are potent ICD inducers, such as chemotherapy (i.e., anthracycline, mitoxantrone, oxaliplatin, and cyclophosphamide), radiotherapy, photodynamic therapy (PDT), and certain oncolytic viruses (i.e., Coxsackievirus B3 and Herpes simplex virus) (85–87).

Translocating pre-apoptotic calreticulin to the cell surface by triggering endoplasmic reticulum (ER) stress and releasing several immune-stimulating factors, such as high mobility group box 1 (HMGB1) and adenosine triphosphate (ATP), which are two processes of ICD (88, 89). For the former pathway, surface-exposed calreticulin binding with the cluster of differentiation 91 (CD91) functions as a “eat-me” signal and recruits phagocytes that uptake dying cancer cells, thereby causing a CTL response in the TME (90). ICD induces cancer cells to release HMGB1, which activates antitumor immunity by enhancing the CD8^+^ T-cell infiltration (91). The HMGB1-toll-like receptor 4 (TLR4) signaling pathway also enhances the antitumor CTL response by promoting the NOD-like receptor family pyrin domain-containing 3 (NLRP3) inflammasome (92). ICD also leads to ATP release by activating autophagy. Extracellular ATP generates a “find-me” signal that recruits monocytes and makes them differentiate into APCs, engulfing dying cancer cells. Extracellular ATP also activates the interleukin 1b (IL-1b) secretion, which is pivotal for a successful CTL activation.

**Figure 3:**
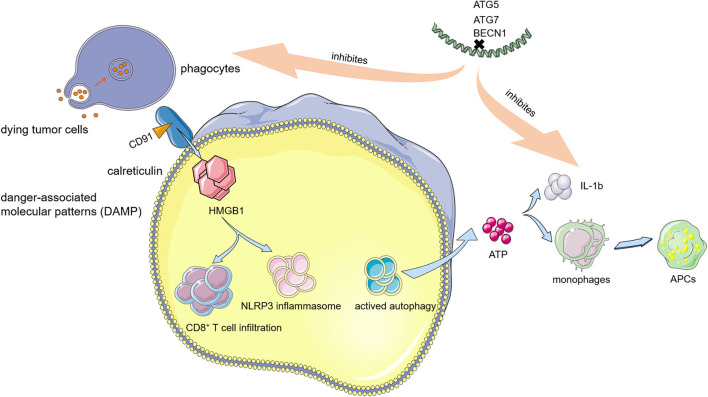
Relationship between immunogenic cell death (ICD) and autophagy. Autophagy modulates the induction of ICD, a non-silent form of cell death, which activates the development of tumor-eradicating cytotoxic T lymphocyte (CTL) response.

Autophagy plays an essential role in inducing ICD because it promotes the release of antigens and danger-associated molecular patterns (DAMPs), such as surface-exposed calreticulin (93). Moreover, another “eat me” signal is also induced by autophagy. In cancer cells, autophagy promotes the release of phosphatidylserine (PS) and facilitates the process of dead cancer cell uptake and tumor antigen presentation (94). Silencing the autophagy genes, such as ATG5, ATG7, and BECN1, could inhibit the DAMP release from mitoxantrone or oxaliplatin-treated cancer cells and subsequently impair the antitumor immunity. In contrast, certain chemotherapeutic agents, such as cisplatin, cannot trigger an ICD due to a lack of stimulation of autophagy in cancer cells (95). In ICD-suffering cancer cells, upon knocking down Beclin-1, ATG5 or ATG7 could reduce the ATP release and consequently inhibit anticancer immunity *in vivo* (Figure 3) (96). The induction of ICD depends on autophagy. Anthracycline treatment cannot affect calreticulin exposure in autophagy-deficient murine colon carcinoma (CT26) cells. Thus, autophagy induces the ICD of cancer cells through the release of ATP and the exposure of calreticulin. Given the aforementioned findings, autophagy promotion synergized with certain kinds of chemo/radiotherapy could be a promising clinical approach through induction of ICD, and related clinical trials are investigated with a combination of autophagy inducers and chemotherapeutics.

## Conclusions and perspectives

Apart from functioning as a critical intracellular bulk degradation system in normal cells as a housekeeper, autophagy also impacts various vital cellular mechanisms in cancer cells. During early tumorigenesis, autophagy is a tumor suppressor, but in advanced cancer stage, its effect remains controversial. There is no doubt that autophagy affects tumor immunity in various ways, including antigen presentation and immune cell development. However, the fundamental machinery of the relationship between autophagy and immunity warrants further elucidation.

Extensive studies demonstrate that autophagy is pivotal for cancer survival and progression (97, 98). Present autophagy-related clinical trials are mainly aimed at the lysosome inhibitors chloroquine (CQ) and hydroxychloroquine (HCQ) (99, 100). In initial clinical trials, patients who received a combinative treatment with either CQ or HCQ and radio- or chemotherapy could have a much better prognosis in various tumors, including advanced solid tumors and hematological malignancies (101–104). However, when more and more clinical trials are carried out, clinical response to autophagy inhibition varies widely. In some clinical trials, the treatment with CQ or HCQ demonstrates no clinical benefit and provides inconsistent evidence of autophagy inhibition (105–107). A major limitation of the current autophagy inhibition treatment has been the identification of appropriate pharmacodynamic biomarkers to evaluate and manipulate the changes in autophagy.

Furthermore, the controversial effect of autophagy is partly owing to the diversity of tumor-infiltrating immune cells in the TME. Autophagy modulates the function of tumor-infiltrating immune cells and regulates their responses to stimuli in the TME. In certain kinds of immune cells, the downregulation of autophagy may facilitate an anticancer response. However, in other tumor-infiltrating immune cells, an increased expression of autophagy could act as an effective anticancer mechanism at various levels. Autophagy inhibition will allow advanced tumors to escape immunosurveillance. For instance, the process of antigen generation and presentation in APCs, especially cross-presentation, deeply relies on autophagy. Autophagy also promotes the proliferation and survival of T cells. In breast cancer, tumors with a higher autophagic flux have more infiltrating CD8^+^ T cells than tumors with a lower autophagic flux (108). Autophagy inhibition represses T-cell proliferation and results in lymphopenia. Furthermore, autophagy inhibition also reduces the related cytokine secretion of T cells, thus reducing the T-cell-dependent cell lysis (108).

Hence, systemic application of autophagy inhibitors does not only perform an antitumor function but also inhibits anticancer immune responses. As a result, the development of therapeutic strategies in cancer based on autophagy inhibition needs more potent and especially more selective drugs. Several new autophagy-targeting drugs, such as VPS34, ARN5187, and Lys05, are in the early stages of investigation (109–111). Their cost performance needs future clinical evaluation. Furthermore, rather than autophagy inhibition in TME, we should also consider activating autophagy to inhibit tumor-promoting inflammation and activate tumor-suppressive immunity. Similarly, autophagy inducers should be delivered into target cells directly and specifically. We propose that target-specific autophagy enhancers could function along with immunogenic chemotherapeutics or immune checkpoint blockade to elevate the efficacy of cancer immunotherapy.

## Author contributions

TY, YZ, and JC performed manuscript drafting. LS supervised the study and edited the manuscript. All authors have read and agreed to the published version of the manuscript.
